# Emerging biomarkers in amyotrophic lateral sclerosis: from pathogenesis to clinical applications

**DOI:** 10.3389/fmolb.2025.1608853

**Published:** 2025-06-30

**Authors:** Farah Anjum, Maha Bakhuraysah, Abdulaziz Alsharif, Taj Mohammad, Anas Shamsi, Md. Imtaiyaz Hassan

**Affiliations:** ^1^ Department of Clinical Laboratory Sciences, College of Applied Medical Sciences, Taif University, Taif, Saudi Arabia; ^2^ King Salman Center for Disability Research, Riyadh, Saudi Arabia; ^3^ Centre for Interdisciplinary Research in Basic Sciences, Jamia Millia Islamia, New Delhi, India; ^4^ Centre of Medical and Bio-Allied Health Sciences Research, Ajman University, Ajman, United Arab Emirates

**Keywords:** amyotrophic lateral sclerosis, molecular basis of neurodegeneration, biomarkers, neuroinflammation markers, therapeutic targets

## Abstract

Amyotrophic lateral sclerosis (ALS) is a severe neurodegenerative condition marked by the gradual loss of motor neurons in the brain and spinal cord. As the most common adult-onset motor neuron disease, ALS manifests through gradually worsening muscle weakness that ultimately progresses to complete paralysis. The disease presents in both sporadic and familial forms. Diagnosis is often delayed until substantial and irreversible motor neuron damage has already occurred. Clinical outcomes in ALS have only been defined through large-scale clinical trials with lengthy follow-up periods due to the disease’s inherent heterogeneity and the absence of disease-specific biomarkers. Current biomarker detection methods, such as invasive cerebrospinal fluid (CSF) analysis or advanced imaging, are impractical for routine use, particularly in late-stage ALS. Several blood-based biomarkers have shown promise, including neurofilament levels, cryptic RNA-derived peptides, and immune-mediated changes, which may enable non-invasive monitoring. Nevertheless, the development of these methods is hindered by technical challenges, such as blood matrix interference and low analyte abundance. Among the emerging biomarkers, neurofilament light chain (NfL) appears to be the most promising, as its concentrations change in line with disease progression and distinguish clinically relevant groups. NfL facilitates patient stratification based on clinical progression rates (e.g., rapid vs slow progressors), while cryptic exon-derived peptides, such as UNC13A-derived peptides, enable genetic stratification by identifying molecular subtypes linked to TDP-43 pathology (e.g., C9orf72 vs sporadic ALS). These biomarkers hold promise to optimize clinical trial design through enriched cohort selection and accelerating therapeutic translation by monitoring target engagement. In this review, we have summarized recent developments in ALS biomarker studies, focusing on neurofilaments in each biofluid, transcriptomic signatures, and neuroinflammatory biomarkers, emphasizing technical challenges surrounding reproducibility in measurement. Finally, we discussed the potential integration of these biomarkers into clinical practice to advance drug development through precision medicine, thereby enabling shorter and more targeted clinical trials.

## 1 Introduction

Amyotrophic lateral sclerosis (ALS), also known as Lou Gehrig’s disease, is a progressive neurological disorder that leads to the deterioration of nerve cells in the brain and spinal cord, ultimately causing muscle weakness and paralysis. ALS has an incidence rate of 2 to 3 per 100,000 worldwide, with a higher prevalence of 6–7 per 100,000 in Europe (Gao et al., 2021). Tragically, most patients survive just 2–5 years after symptom onset, primarily due to complications from respiratory muscle deterioration (Barberio et al., 2023). ALS exhibits marked clinical and genetic heterogeneity, with survival ranging from 2 to over 30 years, and over 30 genes implicated in pathogenesis. This variability highlights the need for biomarkers that address distinct molecular subtypes. For instance, Mutations in SOD1 lead to misfolded protein aggregation in the cytoplasm, contributing to mitochondrial dysfunction (Tafuri et al., 2015), whereas *C9orf72* hexanucleotide repeat expansions drive RNA toxicity via dipeptide repeat protein (DPR) accumulation and nucleocytoplasmic transport defects (Requardt et al., 2021; Schmitz et al., 2021). Typically, initial symptoms manifest between the ages of 55 and 65; however, the disease may also present in elderly individuals or younger populations (Lin et al., 2021). The lack of early diagnostic tools and the average delay of 12–15 months before diagnosis significantly hinder timely intervention. This diagnostic bottleneck highlights the need to integrate biomarkers into clinical workflows.

ALS presents in two primary forms: familial and sporadic (Ajroud-Driss and Siddique, 2015). Familial cases, accounting for about 5%–10%, are associated with inherited genetic mutations, whereas the vast majority, 90%–95%, occur randomly without a clear hereditary pattern (Barberio et al., 2023). Riluzole was historically the sole approved drug, but additional therapies like edaravone and AMX0035 have since been approved in certain regions (Hinchcliffe and Smith, 2017). Non-invasive ventilation is an accepted intervention that enhances the quality of life and survival rates in patients with ALS (Morrison, 2011). Gastrostomy, sialorrhea management, multidisciplinary care, and assistive communication tools effectively improve the quality of life of patients (Ng and Khan, 2012; Garuti et al., 2019). These therapies have limited effectiveness, highlighting the urgent need for biomarkers to enable early diagnosis, guide treatment, and monitor disease progression (De Marchi et al., 2025).

Significant advancements have been made in identifying potential biomarkers for ALS that could support early diagnosis, provide prognostic insights, and deepen understanding of disease progression (Huang et al., 2020). Various targeted approaches, guided by known disease pathways, have been employed to uncover dysregulated molecular signatures. Meanwhile, untargeted approaches, such as large-scale proteomic and lipidomic profiling, have also yielded insights. Similar goals have been achieved by applying targeted methods that systematically examine biomolecules across various domains, including genomics, proteomics, glycomics, and lipidomics techniques (Turner et al., 2009). The biological substances used in the investigation included tissues from patients, encompassing biofluids such as blood, cerebrospinal fluid (CSF), and urine, as well as samples acquired from necropsy, specifically brain and spinal cord tissues. *In vivo* and *in vitro* models of ALS, based on identified mutations linked to the disease, have served as valuable sources for analysis. Different researchers have thoroughly examined various facets of biomarker research for ALS (Turner et al., 2013; Califf, 2018; Xu and Xu, 2024).

Biomarkers typically have multiple, often overlapping purposes (Califf, 2018). Diagnostic biomarkers provide a tool to distinguish between genuine disease and false positives, and have great potential to predict the presentation of disease (Xu and Xu, 2024). In contrast, predictive or prognostic biomarkers can assess the risk of disease progression in patients and the likelihood of patient survival (Sun et al., 2020). Moreover, categorical biomarkers can characterize disease subsets and shed light on disease mechanisms. Clinical pharmacodynamic biomarkers that demonstrate therapeutic efficacy may reduce the need for placebo groups, thereby lowering participant numbers and the duration of clinical trials (Shefner et al., 2022). Recent advances in antisense oligonucleotide (ASO) therapies, such as tofersen (targeting SOD1) and BIIB105 (targeting C9orf72), underscore the critical role of biomarkers in enabling precision medicine (Moriyama and Yokota, 2024). For instance, NfL reduction in CSF correlates with tofersen’s pharmacodynamic effects in SOD1-ALS (Everett and Bucelli, 2024), while poly-glycine-proline (poly-GP) DPR levels serve as target engagement biomarkers in C9orf72 trials (Wilson et al., 2022). However, widespread clinical adoption of these biomarkers requires harmonized assay standardization, cross-platform validation, and multicenter reproducibility studies to address variability in detection methodologies and ensure regulatory compliance (Steinbach et al., 2018; Shefner et al., 2022). In this review, we have examined biomarkers for ALS in the context of emerging technologies and their potential implications for the treatment and management of ALS. Although no biomarker technology has been fully implemented as a primary outcome measure in clinical trials, several are now emerging as valuable adjunct tools, offering complementary support within clinical research and practice.

## 2 Diagnosis of ALS

Electrodiagnostic testing and clinical observation are the primary methods for diagnosing ALS (de Carvalho, 2020). Patients experience a decline in strength and functional capacity over time. A conclusive diagnosis requires clinical evidence of both lower and upper motor neuron (LMN and UMN) involvement to exclude disorders that mimic ALS (Štětkářová and Ehler, 2021). The LMN signs include muscular atrophy, weakness, and superficial fasciculations, while the UMN signs include rapid tendon reflexes, spasticity, and extensor plantar response (Burke, 2021). The presence of fasciculation potentials (e.g., spontaneous electrical activity in muscle fibers) is also essential (Pugdahl et al., 2021). Transcranial magnetic stimulation (TMS) enables the evaluation of UMN involvement (Vucic et al., 2013). Although not yet widely used due to the need for specialized software, threshold tracking TMS has shown promise as a diagnostic tool in the past decade for detecting early signs of increased cortical excitability (Al-Sultan et al., 2019). Advanced neuroimaging techniques are increasingly used to identify reliable and sensitive indicators of upper motor neuron (UMN) lesions in ALS (Bede and Hardiman, 2014). Despite the abundance of possibilities, including magnetic resonance imaging (MRI), spectroscopy, volumetry, functional MRI (fMRI), diffusion-weighted imaging (DWI), and positron emission tomography, further progress is needed to establish a method that is effective for everyday clinical practice. Recent diagnostic innovations include digital biomarkers (e.g., voice/speech analysis, wearable sensors) and AI-driven MRI protocols to detect upper motor neuron involvement (Straczkiewicz et al., 2024).

## 3 Role of genetics in ALS pathogenesis

Genetic contributions are central to ALS development, with multiple genes identified in association with both familial and sporadic forms of the condition (Goutman et al., 2022). Table 1 summarizes the key genes associated with ALS susceptibility and progression (Chia et al., 2018; Mathis et al., 2019). The first gene identified in connection with ALS was superoxide dismutase 1 (SOD1), which encodes a copper-zinc-binding antioxidant enzyme (Wang et al., 2024). It accounts for approximately 20% of familial cases, although there is considerable variation across nations. Notably, disease-causing mutations have been identified throughout the SOD1 protein, not only the metal-binding site (Sirangelo and Iannuzzi, 2017). To date, over 170 unique pathogenic variants of SOD1 have been reported (http://ghr.nlm.nih.gov/gene/SOD1). Such genetic mutations frequently lead to aberrant protein folding and the formation of insoluble SOD1 aggregates, which predominantly accumulate in the cytoplasm of motor neurons (Kaur et al., 2016). These aggregates disrupt normal cellular homeostasis by impairing essential processes such as mitochondrial function, axonal transport, and proteasomal degradation (Magrané and Manfredi, 2009). The resulting cellular stress contributes to motor neuron dysfunction and eventual cell death, hallmark features of ALS pathology (Barber and Shaw, 2010). Beyond SOD1, several other genes linked to RNA and DNA metabolism have emerged as critical in ALS etiology. These genes include *TARDBP, FUS,* and *SETX* (also known as Senataxin). The *TARDBP* gene encodes the TAR DNA-binding protein 43 (TDP-43), which is involved in the majority of sporadic ALS cases (Tziortzouda et al., 2021; Kortazar-Zubizarreta et al., 2023). The (GGGGCC)n repeat expansions (G4C2) in *C9orf72* exert multifaceted effects on cell homeostasis, including disruption of nucleocytoplasmic transport, sequestration of RNA-binding proteins, and translation into DPRs that aggregate and contribute to neurotoxicity (Koppers et al., 2015; McGoldrick and Robertson, 2023). Beyond these genes, various other genetic polymorphisms have been linked to an increased susceptibility to ALS (Table 1).

**TABLE 1:** Key genes associated with amyotrophic lateral sclerosis.

Arrangement	Gene	Localization of chromosome	Proteins and their functions
ALS1	*SOD1*	21q22.11	SOD1: removal of free radicals from the cytoplasm
ALS2	*ALS2*	2q33.2	Alsine: situated on the cytosolic side of endosomes of neurons, with an unclear function
ALS3	*ALS3*		Unidentified function
ALS4	*SETX*	9q32.13	Senataxine: Domain for DNA/RNA helicase
ALS5	*SPG11*	15q14	Spatacsin: Sustaining cytoskeleton and regulating synaptic vesicular transport
ALS6	*FUS*	16p11.2	FUS: Transcription, regulation of splicing, RNA biogenesis, and stress granules formation
ALS7	*ALS7*	20p13	Unidentified
ALS8	*VAPB*	20q13.33	Vesicle-associated membrane protein-associated protein-B (VAPB): Regulates lipid metabolism; manages vesicular transport and the clearance of misfolded proteins (UPR pathway)
ALS9	*ANG*	14q11.1	Angiogenine: trophic factors and angiogenic actors for motor neurons
ALS10	*TARDBP*	1p36.22	TAR DNA binding protein (TDP-43): transcription, splicing, and mRNA transport
ALS11	*FIG4*	6q21	Polyphosphoinositide phosphatase regulates the cell concentration of PI (3,5) P2: This controls endoplasmic vesicle retrograde trafficking to the Golgi
ALS12	*OPTN*	10p13	Optineurine: Membrane transport, cell morphogenesis, vesicular, and transcription activation
ALS13	*ATXN2*	12q23-q24.1	Ataxine-2: interaction with TDP-43
ALS14	*VCP*	9p13	Valosin-containing protein (VCP): ATP transfer via vesicles
ALS15	*UBQLN2*	Xp11.21	Ubiquiline2: degradation of protein
ALS16	*SIGMAR1*	9p13	SIGMAR1 (Sigmanon-opioid intracellular receptor1): Neuro-protective membrane receptor
ALS17	*CHMP2B*	3p12.1	Charged multi-vesicular body protein 2B: Multivesicular bodies (MVBs) are formed
ALS18	*PFN1*	17p13.3	Profiline1: conversion of filamentous actin-(F) from monomeric actin-(G)
ALS19	*ERBB4*	2q33.3-q34	Receptor tyrosine-protein kinase erbB-4: Transcription, cell proliferation, migration, differentiation, and apoptosis
ALS20	*HNRNPA1*	2q13.1	Heterogeneous nuclear ribonucleoprotein A1: Transport of mRNAs to the cytoplasm from the nucleus and splicing modulation
ALS21	*MATR3*	5q31.2	Matrin3: transcription, nuclear retention of defective RNAs, regulation of innate immunity
ALS-FTD2	*CHCHD10*	22q11.23	Coiled-coil-helix-coiled-coil-helix domain-containing protein 10: Preserves the organization and structural integrity of mitochondrial ridges
ALS	*DCTN1*	2p13	Dynactine: the role of dynein in facilitating axonal retrograde transport
ALS-FTD1	*C9ORF72*	9p21	Guanine nucleotide exchange C9orf72: RNA binding and autophagy regulation

The *FUS* gene produces a protein that binds to both RNA and DNA, involved in transcriptional regulation, RNA splicing, and stress granule formation, all vital functions potentially linked to neuronal degeneration in ALS (Deng et al., 2014). The mouse model that expresses the human FUS protein, similar to the one that expresses the TDP-43 protein, exhibits highly aggressive behavior and has a short lifespan (Motaln et al., 2023). The recent identification of a repeated pattern of GGGGCC expansion in a non-coding region of the *C9ORF72* gene has led to its implication in ALS. This could be due to three processes: RNA-binding protein sequestration, dipeptide synthesis (repetition), or haplo-insufficiency (Koppers et al., 2015). Additionally, changes in the *VAPB* gene have been documented in ALS (Borgese et al., 2021). VAPB is involved in vesicle-mediated transport, facilitating the transfer of misfolded proteins during the unfolded protein response (UPR), as well as regulating lipid metabolism and shuttling lipids from the endoplasmic reticulum to other cellular organelles. A Brazilian family was found to carry the VAPB-P56S mutation that binds to the wild-type protein and disrupts its ability to activate the UPR pathway (Kabashi et al., 2013). This dominant-negative effect impairs ER stress responses, contributing to neuronal vulnerability. Additionally, reduced expression of VAPB has been observed in the spinal cords of ALS patients (Anagnostou et al., 2010; Leoni et al., 2022). Other genes involved include *CHMP2B*, *OPTN*, *DCTN1*, *ANG*, *ATXN2*, *UBQLN2*, *PFN1*, and *SQSTM1* (Table 1).

**FIGURE 1:**
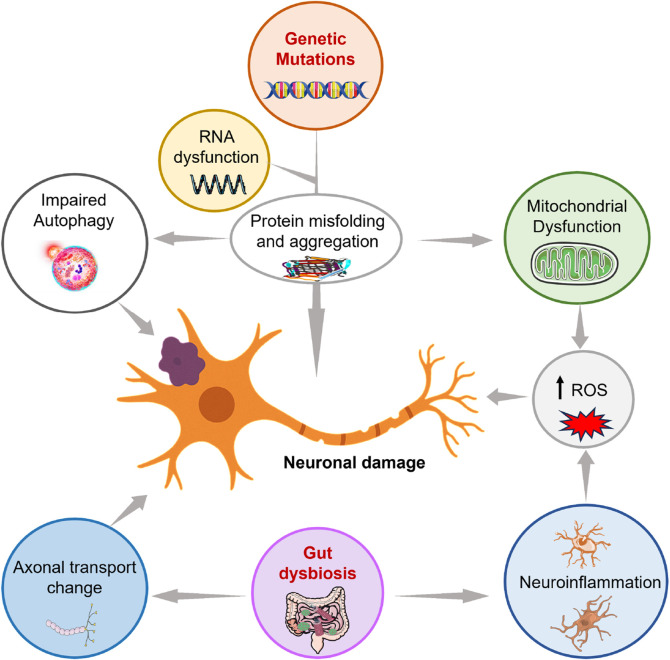
Pathogenic factors and their interactions in amyotrophic lateral sclerosis (ALS). The ALS pathogenesis arises from the interplay of genetic, molecular, and systemic mechanisms. Genetic mutations disrupt RNA processing and protein homeostasis, leading to toxic aggregates that impair mitochondrial function, elevate reactive oxygen species (ROS), and compromise autophagy. Concurrently, defects in axonal transport deprive neurons of critical organelles and proteins, accelerating degeneration. Emerging evidence implicates gut dysbiosis via the gut-brain axis in amplifying neuroinflammation and oxidative stress, further disrupting neuronal integrity. These pathways collectively drive motor neuron loss through interconnected cycles of cellular dysfunction. Preclinical and clinical studies empirically support most of the mechanisms shown, though contributions from gut dysbiosis remain under active investigation. This illustration is inspired by Jiang and Xu (2025).

Extensive research has established that the pathogenesis of ALS arises from a multifactorial interplay of molecular and systemic mechanisms (Taylor et al., 2016; Madji Hounoum et al., 2017) (Figure 1). These include genetic mutations driving RNA misregulation and protein misfolding (e.g., TDP-43, SOD1 aggregates), mitochondrial dysfunction with reactive oxygen species (ROS) overproduction, and impaired autophagy-lysosomal clearance (Jiang and Xu, 2025). Neuroinflammation, mediated by microglial activation and cytokine release, synergizes with these processes to exacerbate neuronal damage. Mitochondrial dysfunction arising from impaired axonal transport deprives motor neurons of ATP, exacerbating ROS accumulation and energy deficits in degenerating axons (Mandal and Drerup, 2019). Concurrently, gut dysbiosis disrupts microbial metabolite production (e.g., short-chain fatty acids), which compromises intestinal barrier integrity and promotes systemic inflammation via gut-derived endotoxin leakage into circulation (Lee et al., 2024). This systemic inflammation exacerbates neuroinflammation through vagus nerve signaling and blood-brain barrier disruption (Anderson, 2022). While mechanistically distinct, these pathways converge to amplify oxidative stress and inflammatory cascades, creating a self-reinforcing cycle of neurodegeneration. Together, these findings highlight the genetic and mechanistic heterogeneity of ALS.

The table summarizes major ALS-linked genes and their chromosomal localizations, as curated from the ALS Online Database (http://alsod.iop.kcl.ac.uk/). The associated proteins and their functional roles in cellular processes relevant to ALS pathophysiology are also listed. UPR: Unfolded Protein Response.

## 4 Biomarkers of ALS

Biomarkers are quantifiable indicators that reflect normal biological functions, the presence of a disease, or the pharmacological response to therapeutic interventions (Califf, 2018). Multiple categories of biomarkers have been recognized, encompassing molecular, radiographic, and physiological markers (Califf, 2018). The NIH-FDA grouped biomarkers into seven separate types, each associated with the BEST (Biomarkers, Endpoints, and other Tools) resources, with an individual role in the pathway to identify biomarkers. These include: (i) susceptibility/risk biomarker, an indicator of a statistical association between a biological characteristic and disease susceptibility; (ii) diagnostic biomarker, an objectively measurable feature reflecting normal function, disease state, or therapeutic response; (iii) monitoring biomarker, a measurable feature used to assess the effectiveness or potential risk of a therapeutic intervention over time; (iv) prognostic biomarker, a characteristic used as a basis for stratified patient therapy to identify those likely to benefit from an intervention; (v) predictive biomarker, a characteristic associated with a defined response; (vi) pharmacodynamic, or response, biomarker, a direct evidence that a biological response has occurred when using an intervention; and (vii) safety biomarker, an indicator of the probability of occurrence, presence, or extent of a toxicity associated with the administration of a therapeutic to a subject. These classifications are integral to advancing therapeutic development, as each provides critical insights that guide the creation of targeted treatment strategies. Neurofilaments, particularly NfL, are among the most clinically advanced biomarkers, validated in multiple ALS cohorts and currently used as secondary endpoints in clinical trials. In contrast, cryptic exon-derived peptides remain in the discovery phase.

### 4.1 Body fluid-based biomarkers

Biomarkers derived from body fluids provide valuable insights into diagnostic, prognostic, and therapeutic responses for ALS (Irwin et al., 2024). These fluid-based biomarkers, detectable in CSF, urine, and blood, include a range of signaling molecules with diagnostic relevance (Table 2). Biomarkers in ALS encompass a diverse spectrum of molecular signatures, including neurofilament isoforms released from degenerating neurons, cryptic peptides arising from aberrant RNA splicing, transcriptional markers indicative of disrupted RNA metabolism, and immune-related alterations that may reflect distinct trajectories of disease progression (McMackin et al., 2023). Researchers in a recent study identified 53 proteins that differed between the CSF samples of individuals with ALS and healthy controls, utilizing a combination of objective discovery-based methods and targeted quantitative comparative analyses after CSF fractionation (Oh et al., 2023). Further analysis of the discovered proteins was then carried out using parallel reaction monitoring (PRM) mass spectrometry. There were notable variations between the ALS and control groups in fifteen proteins: ApoB, APP, CAMK2A, CHIT1, CHI3L1, CLSTN3, FSTL4, ERAP2, GPNMB, JCHAIN, NPTX2, L1CAM, SERPINA3, SERPINA1, and UCHL1.

**TABLE 2:** Detectable fluid biomarkers of ALS in different sources and their detection mode.

Sources	Obstacles to sample collection and analysis	Biological targets and analytes
CSF	Invasive sampling, especially challenging in advanced-stage patients	• Proteins • Mononuclear cells • Small molecules • microRNAs • Antibodies
Urine	Urine concentration and urinary tract infections	• Small molecules
Blood	High protein content interfering with analyte detection (e.g., albumin, immunoglobulins)	• Proteins • Mononuclear cells • Small molecules • microRNAs • Antibodies
Expression of TDP-43 in hiPSCs (human induced pluripotent stem cells)	Low abundance of cryptic peptides in biofluids and technical challenges in mass spectrometry detection	Cryptic peptides

Among the 15 differentially expressed CSF proteins, several have functional relevance to synaptic integrity, axonal structure, and neuronal stress responses (Oh et al., 2023). For instance, CAMK2A and NPTX2 are critical for synaptic plasticity and neuronal excitability, which, when dysregulated, can promote cytoskeletal destabilization and neurofilament disassembly (Arsović et al., 2020; Nugent et al., 2023). CHIT1 and CHI3L1, both associated with neuroinflammation, may reflect microglial activation that facilitates axonal injury and subsequent release of neurofilament subunits into biofluids (Abu-Rumeileh et al., 2020). Notably, proteins such as APP and CLSTN3 are involved in vesicular transport and axonal maintenance, suggesting that their dysregulation could indirectly modulate neurofilament degradation and secretion (Brunholz et al., 2012; Uchida and Gomi, 2016). These associations support a model wherein neurodegenerative processes, captured early by CSF proteomics, culminate in the detectable elevation of neurofilament light chain (NfL) and phosphorylated neurofilament heavy chain (pNfH) in CSF and blood (Alcolea et al., 2023). Thus, the proteomic signatures identified in early-stage CSF profiling may represent upstream mediators or downstream correlates of neurofilament pathology, emphasizing the utility of integrating these markers for early diagnosis and longitudinal monitoring in ALS. Together, these fluid-based biomarkers reflect both axonal injury and neuroinflammatory processes, serving as a foundation for integrating transcriptomic and imaging-based markers.

### 4.2 Neurofilaments

The interplay between neuroinflammatory and proteostatic biomarkers and NfL levels highlights the multifactorial nature of neurofilament release, positioning NfL as a downstream integrator of diverse pathological processes in ALS (Alshehri et al., 2024). Identifying neurofilaments in CSF and blood was an early step in developing fluid-based biomarkers for ALS (Xu et al., 2016). Neurofilament levels are maximum in ALS but are similarly elevated in further neurodegenerative and neuroinflammatory diseases. In presymptomatic ALS mutation carriers, these levels can aid in differential diagnosis and prognosis, including identifying disease onset (Heckler and Venkataraman, 2022). However, it might be oversimplifying to assume that neurofilament release into bodily fluids is directly proportional to the intensity and velocity of axonal degradation (Bridel et al., 2019). A potential therapeutic strategy in ALS involves increasing the expression of NfL, which may shift the balance from the energy-intensive neurofilament heavy chain (NfH) toward smaller, more metabolically efficient subunits. This would help motor neurons conserve energy and respond quickly to pathological situations. As a result, variations in NfL and NfH levels in biofluids may reflect an adaptive response to neurodegeneration rather than a simple indication of loss of axon integrity (Zucchi et al., 2018).

A more nuanced and complex biological phenomenon may underlie the observed neurofilament dynamics in ALS. The humoral immune response, which facilitates the clearance of antigens from circulation to reduce their immune detection, could affect the detectability of neurofilament isoforms in biofluids. Additionally, factors such as body mass index (BMI) have been implicated in modulating neurofilament (Manouchehrinia et al., 2020). Low-dose interleukin-2 (ld-IL-2) selectively expands regulatory T cells (Tregs), which may mitigate neuroinflammation. In the MIROCALS trial, IL-2 therapy reduced CSF pNfH levels, correlating with improved survival, supporting pNfH’s utility as a pharmacodynamic biomarker reflecting treatment response for immunomodulatory interventions (Camu et al., 2020). In a German cohort of SOD1-ALS patients, tofersen therapy reduced CSF and serum NfL levels by 66% and 62%, respectively. However, clinical progression (ALSFRS-R scores) slowed in only two patients, suggesting NfL may serve as a pharmacodynamic biomarker rather than a direct surrogate for clinical efficacy (Meyer et al., 2023).

Phosphorylation of the NfH increases its stability and solubility, impacting its accumulation in axons and detectability in biofluids (Zecca et al., 2022). This modification reflects axonal stress and degeneration, making pNfH a promising candidate for tracking disease progression (Heckler and Venkataraman, 2022). A strong inverse correlation was found between CSF plasma pNfH levels and survival in the MIROCALS trial, which evaluated low-dose IL-2 for the treatment of ALS. This suggests that pNfH may be a useful prognostic marker. By controlling for CSF pNfH levels at randomization, a study on the effectiveness of IL-2 revealed that it significantly improved disease progression and survival (McMackin et al., 2023). Preliminary findings suggest that neurofilaments may serve as biomarkers in clinical trials to identify distinct pharmacological responses, aligning with personalized medicine strategies. Neurofilament levels could serve as a key outcome measure, alongside clinical outcomes, while additional biomarkers would help confirm target engagement. Moreover, some studies on neurofilaments are briefly mentioned in Table 3. Collectively, these studies demonstrate that NfL in blood and CSF is a robust prognostic biomarker, correlating with disease progression and survival.

**TABLE 3:** An overview of neurofilament research and its key findings since 2019.

Biofluid	Methodology	Patient’s cohort	Key findings	References
CSF	ELISA	80 ALS 46 ALS mimics 43 HC	The levels of CSF neurofilament light chain are higher in individuals with ALS than in those with similar conditions and healthy controls	Abu-Rumeileh et al. (2020)
Blood	Simoa	229 ALS 20 PLS 11 progressive muscular atrophy	Baseline serum NfL predicts ALSFRS-R slope. pNfH may serve as a pharmacodynamic biomarker.	Benatar et al. (2020)
Blood	ELISA	382 ALS	Serum NfL concentrations are elevated in women and show a weak correlation with disease progression; higher serum NfL levels are associated with reduced survival	De Schaepdryver et al. (2019)
Blood	Simoa	100 ALS	Serum NfL exhibits a positive correlation with the progression rate. Rapid progressors demonstrate increased median concentrations of NfL and prognostic biomarkers	Dorst et al. (2020)
Blood	ELISA	221 MND	Serum pNfH serves as a negative prognostic indicator for survival. Patients with C9orf72-related motor neuron disease exhibit elevated serum pNfH levels compared to those without C9orf72 mutations	Falzone et al. (2020)
Blood CSF	NfL: confirmed ELISA pNfH: in-house-developed ELISA Simoa	234 ALS 44 ALS mimics 9 controls	CSF NfL and pNfH concentrations are markedly elevated in ALS patients and have a negative correlation with survival. Plasma NfL levels are markedly elevated in ALS patients compared to controls	Behzadi et al. (2021)
Blood CSF	MesoScale Discovery’s R-PLEX Human Neurofilament L Antibody Set	20 ALS 17 IPN	CSF NfL serves as the most reliable indicator of ALS severity. The combination of CSF NfL, CSF ICAM-1, and serum IFN-gamma enhances diagnostic efficacy	Brodovitch et al. (2021)
CSF	Uman Diagnostics’ sandwich enzyme-linked immunoassay	150 ALS 108 HC 28 ALS mimics	CSF NfL demonstrates superior performance compared to hs-cTnT as both a diagnosis and prognostic biomarker	Kläppe et al. (2022)
Blood	Simoa	60 ALS mimics 171 ALS	Plasma and CSF neurofilament light chain levels are significantly elevated in ALS patients compared to mimics, correlate with disease progression and survival, and show stable plasma levels over time	Vacchiano et al. (2021)
Blood	The R-PLEX electrochemiluminescence platform for Meso Scale Discovery	258 ALS 101 HC 80 OND	Plasma NfL correlates with survival outcomes	Thompson et al. (2022)
Blood	Simoa SR-X platform	209 ALS 46 NHC	Serum NfL levels effectively distinguish ALS from NHC, exhibit higher concentrations in females, and correlate with the extent of upper and lower motor neuron involvement. A negative correlation exists between serum NfL and eGFR.	Verde et al. (2023)

### 4.3 Cryptic exons and transcriptomics

**FIGURE 2:**
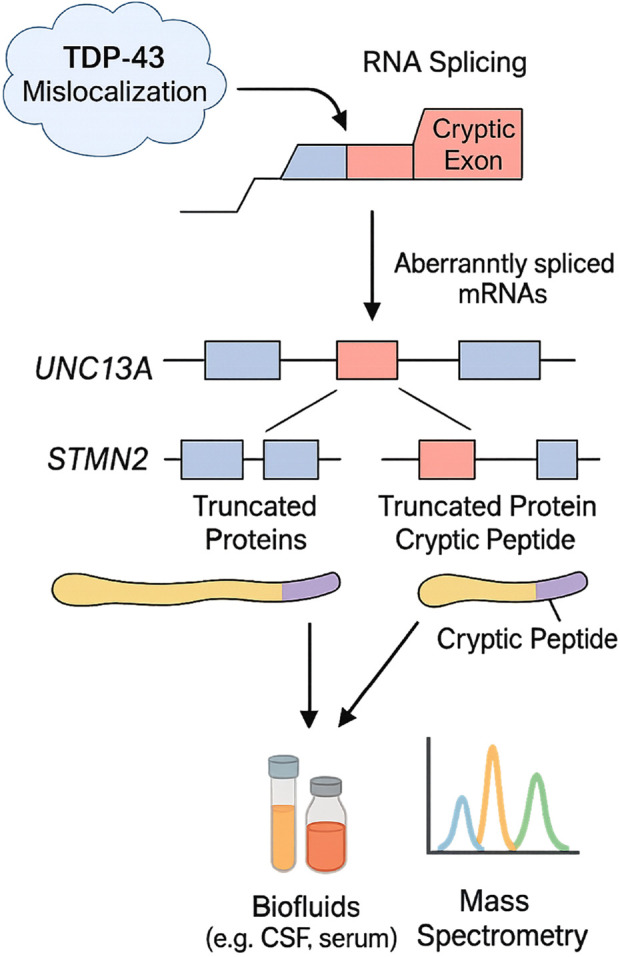
Mechanisms of cryptic exon inclusion in ALS and detection of associated biomarkers. TDP-43 mislocalization disrupts RNA splicing, leading to the incorporation of cryptic exons into transcripts of genes such as UNC13A and STMN2. These aberrantly spliced mRNAs produce truncated proteins with cryptic peptides derived from intronic sequences. Detection of these peptides in biofluids (e.g., CSF, serum) via mass spectrometry offers a novel biomarker strategy for ALS diagnosis and monitoring therapeutic interventions targeting RNA processing in ALS. This schematic integrates both experimentally validated pathways and emerging mechanisms currently under validation in ALS cohorts.

Cryptic exon inclusion has emerged as a hallmark of ALS, particularly through the mislocalization of TDP-43, a nuclear RNA-binding protein essential for splicing regulation (Mehta et al., 2023). Loss of nuclear TDP-43 results in the incorporation of intronic sequences into mature mRNA, creating cryptic exons that disrupt protein-coding sequences (Brown, 2024). UNC13A and STMN2 are notable targets of this mechanism (Mehta et al., 2023). However, TDP-43 is not the sole factor involved; mutations in FUS, another RNA-binding protein, similarly impair splicing fidelity and contribute to generating aberrant transcripts (Rezvykh et al., 2023). These alternative splicing disruptions, whether TDP-43- or FUS-mediated, create novel cryptic peptides that are potential biomarkers for early disease detection and therapeutic monitoring (Akiyama et al., 2022). Given their low abundance and reliance on immunoprecipitation-mass spectrometry, implementing cryptic peptides as routine diagnostic markers remains a technical and economic challenge at present. Loss of TDP-43 function also affects targets like STMN2, leading to the production of aberrant proteins via cryptic exon inclusion (Klim et al., 2019; Mehta et al., 2023). Moreover, oxidative stress plays a critical role in ALS pathogenesis by disrupting redox homeostasis, leading to neuronal damage. Elevated eight-oxo-dG and F2-isoprostanes indicate oxidative DNA and lipid damage. Recent studies highlight TDP-43 and FUS mutations exacerbating oxidative stress via mitochondrial dysfunction (Cunha-Oliveira et al., 2020). Redox imbalance promotes neuroinflammation and motor neuron degeneration. Therapeutic strategies targeting Nrf2 activation or ROS scavengers show promise in preclinical models, underscoring oxidative stress as a key ALS driver (Bozzo et al., 2017). The process and consequences of cryptic exon inclusion, along with detection strategies for cryptic peptides in biofluids, are illustrated in Figure 2.

Consequently, cryptic exon incorporation may provide a range of disease-specific indicators and signal a significant step in the early clinical progression of ALS. Cryptic peptide detection has relied heavily on RNA studies in ALS brain tissue and TDP-43 pathology models in cell lines. Recent efforts to detect cryptic peptides in biofluids, such as UNC13A-derived peptides in CSF and serum, utilize mass spectrometry with immunoprecipitation enrichment (Brown et al., 2022). Preliminary studies report elevated cryptic peptide levels in ALS patients compared to controls, though sensitivity challenges persist due to low abundance (Akiyama et al., 2022). This approach holds significant promise for advancing the discovery of novel fluid-based biomarkers in ALS. If validated, it may enable the development of tools that improve early diagnostic precision. The identification of cryptic peptides, products of TDP-43 dysfunction and primarily associated with ALS and frontotemporal dementia (FTD), offers potential not only as early diagnostic markers but also for stratifying patients based on clinical subtypes (Akiyama et al., 2022). Although they have not yet been widely applied clinically, microRNAs unrelated to plasma cells show promise as biomarkers for ALS prognosis. Similar to NfL, the neuron-specific microRNA miR-181 has been identified in both discovery and replication cohorts as a marker associated with more than twice the risk of mortality in individuals with ALS. The combination of miR-181 and NfL enables the stratification of patient subgroups and the monitoring of disease progression, potentially serving as a predictive biomarker pair comprising RNA and protein (Magen et al., 2021; Liu et al., 2023). Despite their disease specificity, cryptic peptides remain technically difficult to deploy in clinical laboratories due to their low abundance and dependency on immunoprecipitation-coupled mass spectrometry. These platforms require specialized equipment and skilled personnel, limiting current scalability. Efforts toward assay simplification and standardization are ongoing but not yet widely validated.

### 4.4 The immunological response biomarkers

Immune dysregulation is increasingly recognized as a contributing factor in the pathogenesis of ALS, and several biomarkers reflecting immune alterations are under active investigation (Beers and Appel, 2019). Recent studies have focused on soluble substances linked to the innate immune response and macrophage activation, and immune monitoring of ALS disease progression has been achieved through the analysis of cerebrospinal fluid (CSF) and urine. Studies have shown that, compared to healthy controls, ALS patients have higher levels of chitinases in their CSF (Vu et al., 2020). Similarly, p75 and neopterin concentrations in urine are higher in ALS patients and continue to rise as the disease advances (Shepheard et al., 2022). Tregs, characterized by the expression of CD4^+^ and FOXP3^+^, play a crucial role as anti-inflammatory mediators, helping to maintain immune homeostasis and supporting the survival of motor neurons. In ALS, both the number and functional activity of Tregs are significantly reduced as the disease progresses. This decline is strongly associated with increased disease severity and faster progression. As a result, the proportion of circulating Tregs has been proposed as a pharmacodynamic biomarker for monitoring the efficacy of immunomodulatory therapies targeting ALS (Camu et al., 2020) To prevent inflammatory and autoimmune disorders, regulatory T-cells, which are CD4^+^ and FOXP3^+^, play a physiological regulatory role in immune responses (Wing and Sakaguchi, 2010). Tregs may be a therapeutic target for ALS because prior research has linked reduced Treg levels to worse disease severity, illness progression, and survival. Treg function and maintenance are critically dependent on the cytokine interleukin 2 (IL-2) (Chinen et al., 2016). In both mice and humans, the selective expansion of Tregs can be induced by the administration of ld-IL-2 (Zorn et al., 2006). The feasibility and safety of this method have been demonstrated by multiple reported clinical trials that investigated the therapeutic potential of ld-IL-2 in inflammatory and autoimmune disorders (Tahvildari and Dana, 2019).

### 4.5 Functional and metabolic imaging-based markers

Functional and metabolic imaging techniques have become pivotal in detecting early pathophysiological alterations and tracking disease progression in ALS (van den Bos et al., 2019). Resting-state functional MRI studies suggest a gradual decline in connectivity among different brain regions as ALS progresses. In contrast, task-based imaging shows the engagement of atypical brain areas during task execution, likely reflecting compensatory mechanisms. With the development of extra-motor imaging and whole-brain multi-voxel techniques, metabolic imaging methods, such as magnetic resonance (MR) spectroscopy, have also advanced significantly, complementing structural protocols (Christidi et al., 2022; Christidi et al., 2023; McMackin et al., 2023). Additionally, sodium imaging is providing new biological insights (Grapperon et al., 2019). Methods such as motor imaging have been effectively employed, and functional MRI procedures have been more tailored to the impairment profile of ALS patients (Abidi et al., 2022; Münch et al., 2022). Additionally, ALS data sets are being analyzed using texture analysis, which has confirmed patterns of selective vulnerability (Münch et al., 2022). Advanced techniques, such as density imaging and neurite orientation dispersion imaging, have proven helpful in enhancing the understanding of ALS cohorts before symptoms appear (Broad et al., 2019; Bede et al., 2023).

Symptomatic and presymptomatic cohorts have extensively utilized PET imaging to detect early metabolic changes. The creation of specific tracers for astrogliosis and neuroinflammation 103 has demonstrated the use of PET in assessing treatment-related cellular responses (Raval et al., 2023). Recent PET research on ALS has significantly advanced our understanding of phenotype-associated and genotype-associated metabolism-validated presymptomatic changes, as well as potentially adaptive modifications (Canosa et al., 2023; De Vocht et al., 2023). Concurrent PET-MRI modalities offer unique advantages, including improved data integration, refined functional registration, and corrections for regional atrophy, thereby enhancing the interpretability of multimodal data (Costagli et al., 2022; Juengling et al., 2022). By analyzing local changes in susceptibility, quantitative susceptibility mapping can determine the quantities of iron and calcium voxel (Costagli et al., 2022). With its origins as a descriptive tool, quantitative susceptibility mapping is increasingly being utilized in imaging protocols as a biomarker for categorization, diagnosis, and prognosis (Zhang et al., 2023). Recent innovations such as PET-MRI fusion imaging enable high-resolution spatial mapping of metabolic and structural changes, enhancing diagnostic precision (Keir et al., 2024). Furthermore, advanced imaging metrics like texture analysis and quantitative susceptibility mapping (QSM) are under investigation for quantifying iron and calcium accumulation in the motor cortex, changes linked to neurodegeneration in ALS (Mohammadi et al., 2024). When used alongside fluid-based biomarkers like NfL and cryptic peptides, these imaging modalities offer a multidimensional framework for early detection and disease monitoring.

### 4.6 Neuroinflammation-related biomarkers

Neuroinflammation has been recognized as a key feature of ALS pathology, although its role, whether as a consequence of neurodegeneration or as a contributing factor to neuronal loss, remains unresolved (Zhang et al., 2023). In the early stages of ALS, neuroinflammation may help prevent the brain from becoming excessively inflamed and disrupting homeostasis. Immune cells infiltrating from the periphery and reactive microglia in the central nervous system are hallmarks of this phase. Neuroprotective microglia cells become active, anti-inflammatory cytokines are upregulated, and regulatory T cells are enhanced. Activation of neurotoxic microglial cells follows, accompanied by an increase in cytokines and effector T cells, resulting in a proinflammatory response. Although not specific to ALS, these inflammatory markers may support patient stratification, predicting disease progression, and monitoring pharmacodynamic responses in therapeutic trials (Ryberg and Bowser, 2008). Inflammatory situations lead to an upregulation of the chitinases, an enzyme family found in innate immune cells. ALS patients exhibit increased levels of chitinase-like proteins, including chitotriosidase 1 (CHIT1), chitinase-3-like protein 2 (CHI3L2), and chitinase-3-like protein 1 (YKL-40), in contrast to healthy control subjects and individuals with other neurodegenerative disorders (Dreger et al., 2022; Thompson et al., 2022). Elevated CHIT1 levels in CSF reflect macrophage activation and are associated with faster clinical decline, highlighting their role as indicators of neuroinflammatory activity.

Another protein associated with neuroinflammation, particularly astrogliosis, which is seen in ALS patients, is glial fibrillary acidic protein (GFAP) (Verde et al., 2023). The astrocyte cytoskeletal protein with the highest abundance is GFAP. So far, reports on its efficacy as a biomarker have been mixed. Some research has shown a correlation between GFAP levels and the length of time a patient has had ALS or another neurological disorder (Agnello et al., 2021). In response to the production of proinflammatory cytokines, such as IL-1β, IL-6, and TNF-α, hepatocytes generate acute-phase proteins. sCD14, LBP, and C-reactive protein are acute-phase proteins linked to systemic inflammation. Serum levels of all three proteins are known to be elevated in ALS, and sCD14 levels in CSF and urine were found to be higher in ALS patients. Additional validations are required; however, previous research has shown an increase in plasma CK, C3, and serum ferritin, as well as ferritin, creatine kinase (CK), complement C3, and C4 (Shepheard et al., 2022; Thompson et al., 2022). Neopterin is a tiny molecule that is released by monocytic cells in response to IFN-γ. This immune response involves peripheral cells, such as macrophages and dendritic cells, as well as microglia within the central nervous system. Neopterin is another indicator of systemic proinflammation. Therefore, neopterin is a biomarker for cellular-mediated inflammation, and its secretion is associated with ROS generation (Shepheard et al., 2022).

## 5 Therapeutic implications and future directions

Current ALS therapies, including riluzole, edaravone, and AMX0035, provide modest survival or functional benefits (Nikitin et al., 2023). Recent advances in ASOs, such as tofersen (SOD1-ALS) and BIIB105 (C9orf72-ALS), highlight the critical role of genetic biomarkers in patient stratification (Xie et al., 2025). These therapies demonstrate how biomarkers, such as NfL levels and DPR proteins, can be used to track target engagement and predict therapeutic efficacy in real time. Gene-editing approaches (e.g., CRISPR-Cas9 targeting C9orf72 hexanucleotide repeats) and small-molecule modifiers of TDP-43 aggregation are under preclinical investigation (Nowak et al., 2024). Reliable biomarkers are necessary to serve as surrogate endpoints in trials, thereby accelerating drug approval. Immunomodulatory strategies, like low-dose IL-2 to expand regulatory T cells (Tregs), have shown promise in reducing neuroinflammatory biomarkers (e.g., CHIT1, GFAP) and slowing progression in subsets of patients (De Marchi et al., 2023).

Metabolic interventions are also under investigation, including creatine supplementation, NAD + precursors, and agents modulating mitochondrial dysfunction (Lundt and Ding, 2024). These treatments address cellular energy deficits, redox imbalance, and oxidative stress, key contributors to ALS pathogenesis (Sharma et al., 2025). Increasingly, advanced imaging tools such as PET-MRI, sodium imaging, and quantitative susceptibility mapping are being incorporated alongside biofluid biomarkers (e.g., NfL, pNfH, cryptic peptides) to provide multidimensional monitoring of disease progression (Juengling et al., 2022). Integration of imaging and molecular data facilitates precision trial design by enabling real-time stratification of patients into molecular subtypes (Kitaoka et al., 2025).

Initiatives like PRECISION ALS, the ALS CARE Database and the ENCALS (European Network for the Cure of ALS) consortium underscore the importance of large-scale biomarker datasets (McFarlane et al., 2023). AI-driven algorithms now leverage these datasets to identify hidden patient subgroups, optimize trial recruitment, and predict therapeutic response (Kitaoka et al., 2025). Open-access platforms integrating multi-omics data will accelerate the transition from biomarker discovery to clinical translation. Such platforms also enhance reproducibility, enable global collaboration, and reduce the risk of overfitting in biomarker validation. A convergence of high-throughput transcriptomics, proteomics, and digital biomarkers (e.g., voice monitoring, wearable sensors) may yield composite signatures that enable earlier diagnosis and personalized therapy allocation (Kitaoka et al., 2025; Liu and Song, 2025). Combining these emerging strategies with rigorous clinical validation will ultimately be key to transforming ALS management from generalized care to precision neurology.

## 6 Conclusions and prospects

Despite recent advances in identifying numerous candidate biomarkers, none have yet been adopted into standard clinical practice. This reflects the broader understanding that ALS is a heterogeneous disorder, clinically, genetically, and pathophysiologically, making it improbable for any single biomarker to represent the entire disease landscape. Biomarker research has flourished over the past decade, but validation in more extensive clinical datasets, incorporating patient-reported outcomes, remains essential. Detailed clinical phenotyping has brought us closer to elucidate prognostic features, enabling accurate assessment of survival at the group level. Standardized neuropsychological assessments have enabled classification of patients according to whether they exhibit progressive cognitive decline. Concomitantly, genomic studies have begun to characterize disease subtypes by molecular pathway. Still, the earliest diagnosis is a significant problem, often being delayed by approximately 15 months. Despite the limited number of tools for tracking disease progression, scales like ALSFRS-R continue to be crucial for evaluating both early and advanced stages of ALS. The next few years are likely to amplify the paramount importance of understanding ALS variability and, in turn, the biology that underlies it (or *vice versa*). This will most likely include a combination of imaging, fluid-based, and neurophysiological biomarkers. New approaches, such as quantitative EEG, imaging data, and biomarkers derived from cryptic splicing, may improve diagnostic specificity, especially during the presymptomatic stage. The potential for fluid-based markers, such as NfL, in conjunction with advanced neuroimaging and genomic profiling, to facilitate smaller, more focused populations and shorter trial durations could be a game-changer. Additionally, ALS-directed gene product measurements offer pharmacodynamic insights into target engagement within this complex background. Translating these biomarkers into clinical practice will also require standardized assays, regulatory approvals, and infrastructure for routine biomarker testing across healthcare settings. Validated biomarkers could transform ALS research into a precision medicine-driven field, enabling earlier interventions and personalized care. Such a precision approach promises earlier intervention, streamlined trials, and personalized ALS care.
